# Cumulative blood pressure and risk of dementia and cognitive decline: a systematic review and meta-analysis

**DOI:** 10.1016/j.tjpad.2026.100500

**Published:** 2026-02-07

**Authors:** Ruirui Wang, Yijie Gao, Nicole Ee, Fope Akinyede, Xiaoyue Xu, Linan Chen, Shangzhi Xiong, Xiaoying Chen, Craig S. Anderson, Katie Harris, Ruth Peters

**Affiliations:** a Department of Epidemiology, School of Public Health and Jiangsu Key Laboratory of Preventive and Translational Medicine for Major Chronic Non-communicable Diseases, Suzhou Medical College of Soochow University, Suzhou 215123, China; b The George Institute for Global Health, Faculty of Medicine, University of New South Wales, Sydney, NSW 2000, Australia; c School of Population Health, University of New South Wales, Sydney, NSW 2000, Australia; d Institute of Science and Technology for Brain-inspired Intelligence, Fudan University, Shanghai 200032, China; e Neurology Department, Royal Prince Alfred Hospital, Sydney, Australia

**Keywords:** Cumulative blood pressure, Dementia, Cognition, Systematic review, Meta analysis

## Abstract

**Background and objective:**

Cumulative blood pressure (cBP), reflecting long-term BP exposure, is increasingly used to examine risk associations with dementia and cognitive function, but findings to date are inconsistent. This systematic review aimed to synthesize existing evidence to clarify risk associations in adults.

**Methods:**

We searched for articles in Medline, Embase (Ovid), Web of Science, Cochrane Library, and China National Knowledge Infrastructure from inception to January 2025 in any language. Longitudinal, observational studies involving participants aged over 18 years at the time of initial BP assessment were eligible for inclusion. cBP was defined as the area under the curve of BP values over time or an equivalent method, expressed in units of mmHg × time. Study outcomes were dementia, cognitive function assessments, and neuroimaging markers. This review is registered in PROSPERO (CRD42025640637).

**Results:**

From 6334 records identified, 10 independent prospective cohort studies from 9 publications were included in the review, of which four cohort studies were eligible for meta-analysis. Meta-analysis showed that higher cumulative systolic BP (cSBP) was associated with an increased risk of incident dementia (odds ratio [OR] 1.21, 95% CI 1.00–1.45; I² = 92.4%, *P* for heterogeneity<0.001), while cumulative diastolic BP (cDBP) was not associated with dementia risk (OR 0.97, 95% CI 0.72–1.32; I²=97.3%, *P* for heterogeneity<0.001). Among eight studies on cognitive function, five reported that higher cSBP was associated with poorer cognitive performance, while three reported non-significant results. In contrast, findings for higher cDBP were mixed, with two studies reporting adverse associations, two reporting protective associations, and three reporting null associations. Two studies linked higher cSBP and cDBP to greater white matter hyperintensity burden. Sensitivity and subgroup analyses suggested that the positive association between cSBP and dementia-related outcomes were more pronounced among middle-aged adults, whereas inverse or null associations for higher cDBP was observed in some cohorts among individuals aged ≥60 years.

**Conclusion:**

Higher cSBP is associated with increased risk of incident dementia and cognitive decline, whereas associations for cDBP are mixed. Given the limited evidence, future studies should incorporate age-stratified analyses and consider including cumulative pulse pressure and mean arterial pressure to further clarify the relationship between cBP and cognition.

## Graphical abstract

**Figure:**
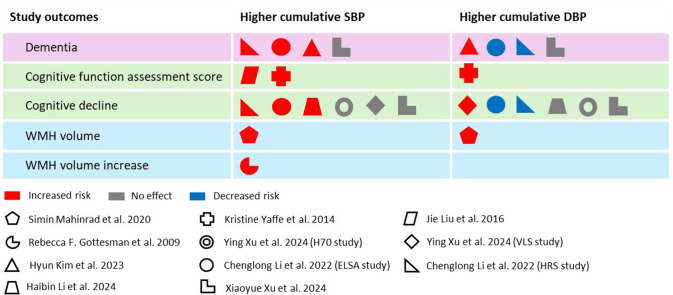

## 1 Introduction

The global prevalence of dementia is rising due to population ageing and dementia prevention has become a key public health priority. In 2020, the number of people with dementia worldwide exceeded 55 million, and this number is expected to double by 2050[1]. Dementia is an irreversible neurodegenerative condition, and despite extensive research efforts, effective therapeutic strategies remain limited[2,3]. Identifying modifiable risk factors and implementing early interventions to reduce the incidence of dementia are current key strategies in addressing this public health challenge.

Higher blood pressure is recognized as a major modifiable risk factor for dementia[4]. Although results from randomized controlled trials remain inconclusive, recent meta-analyses suggest that antihypertensive treatment may have a potential beneficial effect on preventing dementia and cognitive decline [5,6]. The effect of treatment on incident dementia requires long-term observation, with treatment duration and initiation age being important factors to consider[7]. Evidence from population cohort studies is also mixed. Some studies have found that individuals with high BP have a higher risk of dementia[[8], [9], [10]], but others have observed U-shaped associations in older adults[11,12].

BP is a dynamic physiological parameter that exhibits both short-term fluctuations and long-term changes over time. A single time-point BP measurement or the average of multiple readings does not accurately capture an individual's long-term BP burden[13,14]. In recent years, the measurement of cumulative BP has gained increasing attention as this integrates both the intensity and duration of BP exposure. Quantified as the area under the curve and expressed in mmHg*time[14], cumulative BP reflects an individual’s long-term BP exposure thus providing a more comprehensive representation of BP and its potential impact on the structure and function of the cerebral vasculature, including cerebral perfusion and small-vessel integrity, which in turn may contribute to cognitive decline and dementia risk[15].

Recent large-scale epidemiological studies have examined the association between cumulative BP and the risk of dementia and cognitive decline[16,17]. Notably, these studies differ in population characteristics, follow-up durations, and the definition of outcomes. This study therefore aims to systematically review the existing literature and synthesize the evidence to help clarify the relationship between cumulative BP and dementia-related outcomes in adult populations.

## 2 Methods

This study was performed in accordance with the Preferred Reporting Items for Systematic Reviews and Meta-Analyses[18] and registered in PROSPERO (CRD42025640637).

### 2.1 Study selection and search strategy

Medline, Embase (Ovid), Web of Science, Cochrane library, and China National Knowledge Infrastructure (CNKI) were searched from inception to the 16 January 2025 for articles in any language. Details of the search strategy is provided in the **Supplementary Table S1.**

All identified titles and abstracts (or titles alone when abstracts were unavailable) were screened for relevance, and remaining relevant full-texts assessed by at least two independent reviewers (RW, YG, NE, and FA). Conflicts were resolved through discussions until consensus was met.

### 2.2 Inclusion and exclusion criteria

Studies were eligible for inclusion if they met the following criteria: (1) participants were aged over 18 years at the time of initial BP assessment; (2) natural observational studies (cohort or case-control) with a longitudinal design that included baseline and at least one follow-up BP measurement, at least 3 months after baseline; (3) study outcomes assessed at or after the final BP measurement, including clinically diagnosed or self-reported all-cause dementia, dementia defined through cognitive or functional assessment tools, cognitive function assessed using validated scales, or neuroimaging markers; (4) assessed cumulative BP exposure using the area under the curve (AUC) or an equivalent method, expressed in units of mmHg × time. Only full-text, peer-reviewed articles were included; PhD theses, conference abstracts, editorials, and commentaries were excluded.

### 2.3 Data extraction

Data were first extracted by one reviewer (RW), and then independently cross-checked by a second reviewer (YG) using a prespecified extraction form. Extracted information included study population characteristics (sample size, age, sex, ethnic composition, baseline BP level, and use of antihypertensive medications), assessment of cumulative BP, and study outcome. Fully adjusted effect estimates per standard deviation (SD) increase in cumulative BP were extracted where available. If SD-based estimates were not available, the most relevant alternative, such as estimates based on continuous cumulative BP or categorical analyses were extracted. When multiple effect estimates were provided within the same study, the SD-based estimates were prioritized to facilitate comparability across studies.

### 2.4 Quality and bias assessment

Study quality and risk of bias were assessed by two independent reviewers and cross-checked (RW and YG). Study quality was evaluated using a modified version of the Newcastle-Ottawa Scale, which is recommended for observational cohort studies[19]. Potential sources of bias in each study were examined using the Risk Of Bias In Non-randomized Studies of Interventions (ROBINS-I) tool[20].

### 2.5 Data analysis

If multiple studies reported on the identical outcomes from the same cohort, only one was included in subsequent qualitative and quantitative analyses. The selection was guided by a comprehensive evaluation of three key criteria, applied in the following order of priority: (1) overall study quality; (2) availability of comparable effect estimates for the relevant outcomes, in cases where study quality was similar; and (3) sample size, when both quality and effect estimate comparability were equivalent. For any publication that reported results from more than one cohort population, results for each cohort were separately analyzed.

Meta-analyses were conducted using the ‘metafor’ package [21]. All statistical analyses were performed using R version 4.4.2 (R Foundation). Statistical heterogeneity was assessed using the I² statistic, with values between 25 % and 50 % interpreted as moderate heterogeneity, 50 % and 90 % as substantial heterogeneity[22]. Given the clinical and methodological heterogeneity across studies (including differences in sample characteristics, exposure measurements, and outcome definitions), we used a random-effects model as our primary approach.

Subgroup analyses were conducted by age group where possible (≥60 vs <60 years), sex (female vs male), hypertension status (with vs without hypertension), and antihypertensive treatment use (treated vs untreated). All *p* values presented are 2 sided, with a *P* < 0.05 considered statistically significant.

## 3 Results

**Fig. 1:**
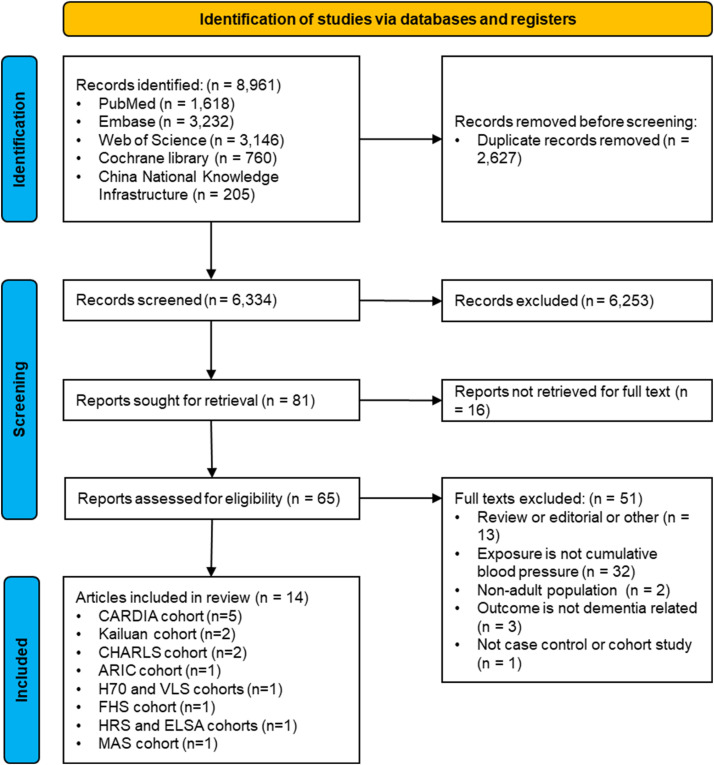
**Screening flow chart**. ARIC, Atherosclerosis Risk in Communities; CARDIA, Coronary Artery Risk Development in Young Adults; CHARLS, The China Health and Retirement Longitudinal Study; ELSA, English Longitudinal Study of Ageing; FHS, Framingham Heart study; H70, Gothenburg H70 Birth Cohort; HRS Health and Retirement Study; MAS, The Sydney Memory and Aging Study; VLS, Victoria Longitudinal Study.

As shown in Fig. 1, 6334 records were identified through database searches. Among the 81 records assessed at the full-text stage, 67 were deemed ineligible. Sixteen were excluded due to unavailability of full texts (14 abstract-only publications, 1 inaccessible record from a German database, and 1 conference poster) and 51 records did not meet the eligibility criteria. A full list of reasons for exclusion is detailed in **Supplementary Table S2.** This resulted in 14 full-text articles that met the inclusion criteria[16,17,[23], [24], [25], [26], [27], [28], [29], [30], [31], [32], [33], [34]].

Ultimately, we included 10 independent population-based prospective cohort studies from 9 publications in our qualitative and quantitative analyses[16,17,25,27,[29], [30], [31], [32],^34^]. Since multiple studies reported on identical outcomes from the same cohort, the prioritization criteria detailed above was employed to select respective results per cohort. Five articles reported data from the CARDIA cohort[16,[23], [24], [25], [26]]. Of these, four examined the association between cumulative BP and cognitive function assessments[16,[23], [24], [25]], and two reported outcomes related to WMH volume[16,26]. Cognitive function results from Kristine Yaffe et al.[25]. was included in the analysis, as it comprised of the largest sample size. For WMH, Simin et al.[16]. was selected, as the other study was a mediation analysis[23]. Two articles reported results from the Kailuan cohort on cognition[27,28]. The results from Jie et al.[27]. was preferred because it reported effect sizes per SD of cumulative BP, expressed as beta (95 % CI), which allowed for comparability with other studies, and also had the larger sample of the two. Similarly, two articles reported results from the CHARLS cohort[32,33] on cognition and Haibin et al.[32]. was preferred for comparability of effect estimates.

### 3.1 Characteristics of the included studies

The included studies were conducted in general population cohorts from the United States[16,17,25,29,31], China[27,32], Sweden[30], Canada[30], the United Kingdom[17], and Australia [34]. Among the 10 independent prospective cohort studies, one cohort enrolled participants aged 18–30 years at baseline[16,25], four cohorts focused on middle-aged groups[27,29,31,32], and five cohorts were based on older populations, with a median baseline age over 60 years[17,30,34]. All included studies involved both male and female participants. At baseline, participants had a mean systolic BP (SBP) of <140 mmHg in 8 cohorts[16,17,25,[29], [30], [31], [32],^34^], while mean SBP was ≥140 mmHg in the H70 cohort[30] and the ELSA cohort[17]. The characteristics of the included studies are summarised in Table 1.

**Table 1:** Characteristics of the included studies.

Number	Study	Study Country	Data source	Sample size	Study population	BP measures	Cumulative BP measures	Follow-up	Study outcome
1	Simin Mahinrad et al. 2020*	U.S.	CARDIA cohort	*N* = 191, and *N* = 144 for WMH data	(1) Adults aged 18 to 30 years at baseline; mean baseline age 24 ± 4 years;(2) 46 % female;(3) 56 % White and 44 % Black; (4) Baseline SBP=108±10 mmHg; and antihypertensive medication use less than 30 %.	Sitting, automatic electronic BP monitor	AUC, examined from baseline after 2, 5, 7, 10, 15, 20, 25, and 30 years.	Study outcome was assessed at the 30-year follow-up.	(1) Cognitive function assessment: executive, memory, attention, and global cognitive function. The tests included the Hopkins Verbal Learning Test- Revised, the Digit Span test, the Trail Making Test A and B, the Digit Symbol Substitution Test (DSST), the Stroop test, and the National Institute of Health Flanker Inhibitory Test, List Sorting Working Memory Test, and Picture Sequence Memory Test. The higher composite score indicated better cognitive performance. (2) White Matter Hyperintensity (WMH)
2	Lisanne M. Jenkinsa et al. 2021	U.S.	CARDIA cohort	*N* = 578	(1) Adults aged 18 to 30 years at baseline;(2) 56 % female;(3) 58 % White and 42 % Black.	Sitting, automatic electronic BP monitor	AUC, examined from baseline after 2, 5, 7, 10, 15, 20, 25, and 30 years.	Study outcome was assessed at the 30-year follow-up.	(1) Cognitive function assessment: the Stroop test. Higher scores indicate worse performance.
3	Christina S. Dintica1 et al. 2022	U.S.	CARDIA cohort	*N* = 661	(1) Adults aged 18 to 30 years at baseline; mean baseline age 25.3 ± 3.5 years;(2) 52.1 % female;(3) 60.0 % White and 40.0 % Black; (4) Baseline SBP=110±10.3 mmHg.	Sitting, automatic electronic BP monitor	Time-weighted averages of BP were calculated as = the AUC divided / time interval. AUC, examined from baseline after 2, 5, 7, 10, 15, 20, 25, and 30 years.	Study outcome was assessed at the 30-year follow-up.	(1) Cognitive function assessment: Including the Digit Symbol Substitution Test, the Stroop Test, the RAVLT, and verbal fluency. The higher composite score indicated better cognitive performance.
4	Kristine Yaffe et al. 2014*	U.S.	CARDIA cohort	*N* = 3381	(1) Adults aged 18 to 30 years at baseline; mean baseline age 25.1 ± 3.6 years;(2) 56.4 %female;(3) 53.6 % White and 46.4 % Black; (4) Baseline SBP=109±10.8 mmHg.	Sitting, automatic electronic BP monitor	AUC, examined from baseline after 2, 5, 7, 10, 15, 20, and 25 years.	Study outcome was assessed at the 25-year follow-up.	(1) Cognitive function assessment: the Digit Symbol Substitution Test, the Stroop Test, and the RAVLT. The higher score indicated better cognitive performance.
5	Lisanne M. Jenkinsa et al. 2020	U.S.	CARDIA cohort	*N* = 144	(1) Adults aged 18 to 30 years at baseline; mean baseline age 25. ± 35 years;(2) 56.4 %female;(3) 53.6 % White and 46.4 % Black; (4) Baseline SBP=109±10.8 mmHg.	Sitting, automatic electronic BP monitor	AUC, examined from baseline after 2, 5, 7, 10, 15, 20, 25, and 30 years.	Study outcome was assessed at the 30-year follow-up.	(1) WMH volume
6	Jie Liu et al. 2016*	China	Kailuan cohort	*N* = 2211	(1) Adults aged over 40 years at baseline, mean baseline age 51.04 ± 10.56 years;(2) 41.4 % female; (3) All Chinese population; (4) Baseline SBP=125.96±18.79 mmHg; and proportion of antihypertensive treatment was 10.72 %.	Sitting, calibrated mercury sphygmomanometer	AUC, cumulative SBP = [(SBP2006 + SBP2008) /2 × time2008–2006] + [(SBP2008 + SBP2010) /2 × time2010–2008] + [(SBP2010 + SBP2012) /2 × time2012–2010].	Study outcome was assessed at the fourth follow-up examination from 2012 to 2013.	(1) Cognitive function assessment: Mini-Mental State Examination (MMSE). The higher score indicated better cognitive performance.
7	Huijing Shi et al. 2023	China	Kailuan cohort	*N* = 1045	(1) Adults aged over 40 years at baseline, mean baseline age 43.1 ± 9.9 years;(2) 49.4 % female; (3) All Chinese population; (4) Baseline SBP=117.8 ± 16.5 mmHg; and proportion of antihypertensive treatment was 8.8 %.	Sitting, calibrated mercury sphygmomanometer, from the fourth follow-up (2014) onwards, using a digital BP monitor.	AUC, cumulative SBP = [(SBP2006 + SBP2008) /2 × time2008–2006] + [(SBP2008 + SBP2010) /2 × time2010–2008] + ...… + [(SBP2018 + SBP2020) /2 × time2020–2018])	Study outcome was assessed at seventh follow-up between 2020 and 2018.	(1) Cognitive function assessment: Montreal Cognitive Assessment. The higher score indicated better cognitive performance.
8	Rebecca F. Gottesman et al. 2009*	U.S.	ARIC cohort	*N* = 983	(1) Middle-aged adults;(2) 61.6 % female;(3) 49.3 % Black and 50.7 % White; (4) Baseline SBP=125.96±10.56 mmHg.	Sitting, automatic electronic BP monitor	AUC/ time, from the baseline visit 1987–1989, 1990 to 1993, 1993 to 1995, 1996 to 1999, and 2004 to 2006.	Study outcome was assessed between 1993–1995 and 2004–2006.	(1) WMH change Progression of WMHs between visit 3 and visit 5
9	Ying Xu et al. 2024*	Sweden and Canada	H70 and VLS cohort	H70: *N* = 604; VLS: *N* = 557	(1) Aged over 70. Mean baseline age 70.6 ± 0.3 years in H70 and 77.6 ± 4.9 in VLS;(2) 59.8 % female in H70 and 61.4 % female in VLS; (3) Baseline SBP= 154.9 ± 22.2 mmHg in H70 and 131.6 ± 15.1 mmHg in VLS; proportion of antihypertensive treatment was 83.4 % in H70 and 27.1 % in VLS.	Sitting, automatic electronic BP monitor	Cumulative BP=BP1*(time2-time1) + BP2*(time3–2) + BP3.(1) H70: 2000–2002,2005–2007 and 2009–2011. Mean (standard deviation) follow-up was 8.5 (2.0) years(2) VLS: 1998–2002, and approximately 4 yearly intervals thereafter. Mean (standard deviation) follow-up was 8.5 (4.0) years in the VLS.	Assessed at the baseline and last available follow-up.	(1) Cognitive function assessment H70: MMSE, and cognitive tests including immediate and delayed word recall from the Alzheimer’s Disease Assessment Scale-Cognitive and of semantic verbal fluency task. VLS: MMSE and cognitive tests including the RAVLT (List A, trials 1–5; List B and List A, trial 6), and the Wechsler Adult Intelligence Scale-Revised Digit Symbol Substitution task. Cognition change was calculated by subtracting the baseline score from the follow-up score such that a negative score indicated a decrease in cognitive performance.
10	Hyun Kim et al. 2023*	U.S.	FHS cohort	*N* = 3201	(1) Adults aged between 40–64 years, mean baseline age 45.91±2.37 years;(2) 55.23 % female;(3) 100 % Non-Hispanic Whites; (4) Baseline SBP= 123.74±16.66 mmHg; and proportion of antihypertensive medication was 6.10 %.	Sitting, calibrated mercury sphygmomanometer	AUC, five examinations spanning up to a 22-year observation period. Median BP observation duration was 16.39 years.	The follow-up window was up to 42 years (median 17 years).	(2) All-cause dementia Criteria from the Diagnostic and Statistical Manual of Mental Disorders, 4th edition were used to define dementia.
11	Chenglong Li et al. 2022*	U.S. and U.K.	HRS and ELSA cohort	*N* = 7566 in ELSA, *N* = 9294 in HRS	(1) Aadults over 50 years of age. Median (IQR) baseline age 62.0 (55.0–70.0) years in ELSA and 65.0 (58.0–72.0) in HRS;(2) 55.2 % female in ELSA and 59.8 % female in HRS;(3) 98.3 % White in ELSA and 83.2 % White in HRS; (4) Baseline SBP= 141.3 ± 19.5 mmHg in ELSA and 129.8 ± 19.7 mmHg in HRS; and proportion of antihypertensive medication was 27.0 % in ELSA and 46.7 % in HRS.	Sitting, automatic electronic BP monitor	AUC (1) In ELSA, BP measurements from wave 0 (1998), wave 2 (2002), and wave 4 (2008) were used to evaluate cumulative BP. (2) In HRS, BP measurements from wave 8 (2006) and wave 10 (2010) were used to evaluate cumulative BP.	(1) For ELSA, cognitive decline and dementia were assessed from wave 4 (2008) to wave 9 (2018), median follow-up 8.0 years. (2) For the HRS, cognitive decline and dementia were assessed from wave 10 (2010) to wave 14 (2018), median follow-up 8.0 years.	(1) Cognitive function assessment: Immediate and delayed word recall tests were administered to assess memory and the date-naming test to assess orientation. For executive function, tests including counting backward and the serial-sevens test were implemented in the HRS, while the animal-naming fluency test was implemented in ELSA. For all tests, a higher score indicated better cognitive performance.(2) Dementia cases using either a self-reported physician diagnosis or an alternative approach based on cognition and functionality scores. For the HRS, a cognition summary score ranging from 0 to 27, with a cutoff point of 6 or less defined as dementia. For ELSA, dementia as the coexistence of cognitive and functional impairment. Cognitive impairment was defined as a score that was 1.5 SDs lower than the mean of the population stratified by educational background. Functional impairment was defined as difficulty in performing 1 or more activities of daily living, including bathing, eating, dressing, getting into and out of bed, and walking across a room.
12	Haibin Li et al. 2024*	China	CHARLS cohort	*N* = 7925	(1) General population without cognitive impairment aged 45 years and older;(2) 52.7 % female; (3) All Asian; (4) Baseline SBP>140/90 mmHg or taking anti-antihypertensive medication use is 37.1 %*(in the whole population of 11,671 participants).	Sitting, automatic electronic BP monitor	AUC, measured from wave 1 (2011) and wave 2 (2013).	Cognitive decline was assessed from wave 2 (2013), wave 3 (2015), and wave 4 (2018).	(1) Cognitive Function Assessment First, mental intactness was assessed based on components of the Telephone Interview of Cognitive Status battery, which included performing serial 7 subtraction from 100, naming today’s date, and testing the ability to redraw a picture. Second, episodic memory was assessed by immediate and delayed word recall tasks. Global cognitive scores were calculated as the sum of the scores of episodic memory and mental intactness and ranged from 0 to 21.
13	Lili Luo et al. 2024	China	CHARLS cohort	*N* = 10,366	(1) General population without cognitive impairment aged 45 years and older;(2) 54.5 % female; (3) All Asian.	Sitting, automatic electronic BP monitor	AUC, measured from wave 1 (2011–2012), wave 2 (2013–2014), and wave 3 (2015–2016).	The cognitive function was assessed at wave 4 (2017–2018).	(1) Cognitive Function Assessment First, mental intactness was assessed based on components of the Telephone Interview of Cognitive Status battery, which included performing serial 7 subtraction from 100, naming today’s date, and testing the ability to redraw a picture. Second, episodic memory was assessed by immediate and delayed word recall tasks.
14	Xiaoyue Xu et al. 2024*	Australia	MAS cohort	*N* = 1037	(1) Adults aged 70–90 years;(2) 54.5 % female; (3) All Asian; (4) Baseline SBP mean value ranges from 127 (15) to 162 (17); proportion of antihypertensive medication ranges from 42.9 % to 68.4 %.	Sitting, automatic electronic BP monitor	AUC, measured from 2005 to 2007, and two waves about two-yearly intervals followed by baseline examination.	(1) Cognitive scores were derived from waves 1 (2005–2007) and 4 (2012–2014), and data from waves 3 and 4 were used to capture cognitive decline. (2) Dementia cases: over a mean of 10.5 (SD= 2.6) years follow-up since wave 3.	(1) Cognitive Function Assessment Trained psychology graduates administered a comprehensive neuropsychological test battery covering 5 major cognitive domains: attention/processing speed (Trail Making Test A, Digit-Symbol Coding; language (Boston Naming Test (30-item), Category Fluency (Animals); memory (Benton Visual Retention Test (Recognition), Logical Memory Story A delayed recall, RAVLT (Total learning, Immediate recall and delayed recall); executive function (Letter fluency (FAS), Trail Making Test B; visuospatial (Block Design). (2) Dementia Dementia was diagnosed according to the criteria outlined in the Diagnostic and Statistical Manual of Mental Disorders, Fourth Edition and required deficits in at least 2 cognitive domains, including memory, and impairment in activities of daily living.

⁎ Studies included in the analysis. AUC, area under the curve (mmHg*time); BP, blood pressure; SBP, systolic blood pressure; RAVLT, Rey Auditory Verbal Learning Test; IQR, interquartile range. ARIC, Atherosclerosis Risk in Communities; CARDIA, Coronary Artery Risk Development in Young Adults; CHARLS, The China Health and Retirement Longitudinal Study; ELSA, English Longitudinal Study of Ageing; FHS, Framingham Heart study; H70, Gothenburg H70 Birth Cohort; HRS Health and Retirement Study; MAS, The Sydney Memory and Aging Study; VLS, Victoria Longitudinal Study.

### 3.2 Cumulative BP assessment

All included studies reported the procedures used for BP measurement and all cohorts used sitting BP values. Eight cohorts used automated electronic BP monitors[16,17,25,29,30,32,34], and two cohorts used calibrated mercury sphygmomanometers[27,31]. Assessment of cumulative BP was calculated using the AUC method in seven cohorts[16,17,25,27,31,32,34], using a 'pack-year'–like calculation method in the H70 and VLS cohorts [30], and as AUC divided by time in the ARIC cohort [29]. The duration of cumulative BP measurement varied across studies: three cohorts assessed BP over a period of >10 years[16,25,29,31]; four cohorts had cumulative BP durations of 5–10 years[17,27,30,34]; and three cohorts measured BP over a period of 2–5 years[17]^,^ [32].

### 3.3 Outcome assessment

Two cohorts identified dementia cases using clinically diagnosed criteria[31,34], the other two cohorts[17] defined dementia either through self-reported physician diagnosis or via alternative approaches based on cognitive functional assessment scores[[35], [36], [37]]. The cognitive function assessment varied in both the scales applied and the cognitive domains evaluated. Composite cognitive tests or scores were reported in eight cohorts^25,27,30, 17,^ [32,34]. Some studies also reported cognitive tests results by specific domains. Memory function was assessed in six cohorts[17,25,30,32]; executive function was evaluated in three cohorts[17,25]; orientation was assessed in two cohorts[17]; verbal fluency was measured in the H70 cohort[30]; and mental intactness was assessed in the CHARLS cohort[32]. Additionally, two cohorts measured WMH volume—one cohort assessed this at the final follow-up[16], while the other cohort assessed the change in WMH volume between two follow-up time points[29].

### 3.4 Meta analysis for the association between cumulative BP and dementia

**Fig. 2:**
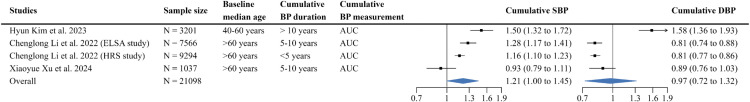
**Odds ratios (95 % confidence interval) for the associations between per SD increase in cumulative BP and risk of dementia**. AUC, area under the curve (mmHg*time); SBP, systolic blood pressure; DBP, diastolic blood pressure. Cumulative SBP: *P* for heterogeneity < 0.001, I^2^=92.36 %; τ[2]=0.032. Cumulative DBP: *P* for heterogeneity < 0.001, I^2^=97.31 %; τ[2]=0.095.

A total of four cohorts reported the association of per 1-SD increase of cumulative BP on dementia risks[17,31,34]. Overall, each 1-SD increase in cumulative SBP was associated with an increased odd of dementia (OR 1.21, 95 % CI 1.00–1.45). However, there was substantial heterogeneity across studies (I²= 92.36 %, *τ*^2^=0.032, P for heterogeneity < 0.001) (Fig. 2). As a sensitivity analysis, when we excluded one cohort study[31], which involved a younger population compared to the other three cohorts. This resulted in the association between cumulative SBP and dementia being no longer statistically significant (OR 1.13, 95 % CI 0.95–1.34; I²= 89.07 %, *τ*^2^=0.019, P for heterogeneity =0.005) (**Supplementary Table S3**).

For cumulative diastolic BP (DBP), each 1-SD increase was not significantly associated with dementia in the meta-analysis (OR 0.97, 95 % CI 0.72–1.32; I² = 97.31 %, *τ*^2^=0.095, P for heterogeneity < 0.001) (Fig. 2). However, after excluding the cohort with younger participants[31], each 1-SD increase in cumulative DBP was associated with a reduced odds of dementia (OR 0.82, 95 % CI 0.78–0.85; I² = 0.05 %, *τ*^2^=0, P for heterogeneity = 0.510) (**Supplementary Table S3**).

### 3.5 Cumulative BP and cognitive function

Given the substantial differences in cognitive instruments (global vs domain-specific), outcome definitions (cognitive level vs decline), and effect metrics across studies, we summarised the cognitive findings narratively rather than pooling them in a meta-analysis.

Among the eight cohort studies that assessed the association between cumulative SBP and composite cognitive scores, five cohorts[17,25,27,32] reported a negative association; H70 and MAS cohorts[30]^,^ [34] found no association; and VLS cohort [30] reported a negative association in a multi-domain cognitive assessment, but not with MMSE score. Among the six cohorts that assessed cumulative SBP and memory function independently, four cohorts[17,25,32] reported a negative association; H70 cohort[30] found no association. In the VLS study[30], higher cumulative SBP was associated with increased decline in immediate recall (Rey Auditory Verbal Learning Test [RAVLT] List A, trials 1–5), but not associated with free recall (RAVLT List B) or delayed recall (RAVLT List A, trial 6). Among the three cohorts that assessed cumulative SBP and executive function, the CARDIA and ELSA cohorts [17,25] reported a negative association, while the HRS cohort[17] reported no association. For the association between cumulative SBP and orientation function, two cohorts provided data: the ELSA study[17] reported a negative association, whereas the HRS study[17] reported no association. Additionally, higher cumulative SBP was associated with decline in mental intactness (based on components of the Telephone Interview of Cognitive Status) in CHARLS cohort[32], but not for verbal fluency in H70 cohort[30]. (Table 2)

**Table 2:** Beta coefficients (95 % CI) for the associations between higher cumulative BP and cognitive decline and white matter hyperintensities (WMH).

Studies	Sample size	Baseline median age	Cumulative BP duration	Cumulative BP measurements	Cumulative SBP	Cumulative DBP
**Cognitive function assessment score**						
**Kristine Yaffe et al. 2014**^a^	*N* = 3381	<40 years	> 10 years	AUC		
Attention, working memory, psychomotor speed, and executive function (Digit Symbol Substitution Test)					−0.12 (−0.18, −0.06)	−0.07 (−0.12, −0.02)
Executive (Stroop Test)					−0.11 (−0.17, −0.05)	−0.09 (−0.14, −0.03)
Verbal memory (RAVLT)					−0.09 (−0.15, −0.03)	−0.05 (−0.11, 0.00)
**Jie Liu et al. 2016**^a^	*N* = 2211	40–60 years	5–10 years	AUC		
MMSE					−0.36 (−0.52, −0.21)	-
**Cognitive decline**						
**Ying Xu et al. 2024 (H70 study)**^b^		>60 years	5–10 years	Pack year*		
Immediate recall (ADAS-Cog)	*N* = 445				0.07 (−0.0003, 0.13)	0.09 (−0.04, 0.22)
Delayed recall (ADAS-Cog)	*N* = 304				−0.13 (−0.36, 0.09)	−0.02 (−0.51, 0.46)
Verbal fluency (Semantic verbal fluency tasks)	*N* = 445				−0.16 (−0.31, 0.00)	−0.33 (−0.62, −0.03)
MMSE	*N* = 438				−0.02 (−0.11, 0.07)	0.01 (−0.17, 0.19)
**Ying Xu et al. 2024 (VLS study)**^b^		>60 years	5–10 years	Pack year*		
Immediate recall (RAVLT List A, trials 1–5)	*N* = 328				−0.23 (−0.32, −0.13)	−0.41 (−0.58, −0.23)
Free recall (RAVLT List B)	*N* = 108				−0.01 (−0.09, 0.07)	−0.004 (−0.16, 0.15)
Delayed recall (RAVLT List A, trial 6)	*N* = 108				−0.03 (−0.17, 0.12)	−0.02 (−0.30, 0.25)
Attention, information processing speed, and visuospatial scanning (WAIS-R DSS)	*N* = 247				−0.59 (−0.80, −0.38)	−1.04 (−1.40, −0.67)
MMSE	*N* = 358				−0.04 (−0.09, 0.01)	−0.09 (−0.17, −0.01)
**Chenglong Li et al. 2022 (ELSA study)**^a^	*N* = 7566	>60 years	5–10 years	AUC		
Memory (Immediate and delayed word recall tests)					−0.008 (−0.011, −0.004)	0.004 (0.001, 0.008)
Executive (Counting backward and the serial-sevens test)					−0.007 (−0.010, −0.003)	0.004 (0.000, 0.008)
Orientation (Date-naming test)					−0.008 (−0.012, −0.003)	0.005 (0.001, 0.010)
Composite score					−0.013 (−0.017, −0.009)	0.007 (0.003, 0.011)
**Chenglong Li et al. 2022 (HRS study)**^a^	*N* = 9294	>60 years	<5 years	AUC		
Memory (Immediate and delayed word recall tests)					−0.006 (−0.009, −0.003)	0.005 (0.002, 0.008)
Executive (Counting backward and the serial-sevens test)					0.000 (−0.003, 0.003)	0.005 (0.002, 0.008)
Orientation (Date-naming test)					−0.007 (−0.015, 0.001)	0.001 (−0.006, 0.009)
Composite score					−0.009 (−0.012, −0.005)	0.009 (0.005, 0.012)
**Haibin Li et al. 2024**^a^	*N* = 7925	40–60 years	<5 years	AUC		
Mental intactness (Telephone Interview of Cognitive Status)					−0.005 (−0.009, −0.001)	−0.001 (−0.006, 0.003)
Episodic memory (Immediate and delayed word recall tasks)					−0.017 (−0.024, −0.010)	0.002 (−0.005, 0.009)
Composite score					−0.012 (−0.017, −0.007)	−0.001 (−0.006, 0.003)
**Xiaoyue Xu et al. 2024**^a^	*N* = 1037	>60 years	5–10 years	AUC		
Composite score (Attention/processing speed, language, memory, executive function and visuospatial ability)					−0.04 (−0.14, 0.06)	−0.01 (−0.10, 0.08)
**WMH volume**						
**Simin Mahinrad et al. 2020**^a^	*N* = 144	40–60 years	> 10 years	AUC	0.31 (0.06, 0.56)	0.25 (0.006, 0.5)
**WMH volume increase**						
**Rebecca F. Gottesman et al. 2009**^c^	*N* = 983	40–60 years	> 10 years	AUC/time	2.35 (1.58, 3.12)	-

ADAS-Cog, Alzheimer’s Disease Assessment Scale-Cognitive; AUC, area under the curve (mmHg*time); BP, blood pressure; SBP, systolic blood pressure; DBP, diastolic blood pressure; MMSE, Mini-Mental State Examination; RAVLT, Rey Auditory Verbal Learning Test; WAIS-R DSS, Wechsler Adult Intelligence Scale-Revised Digit Symbol Substitution task.

a Effect sizes represent associations per 1-SD increase in cumulative BP;.

b Effect sizes represent associations per 100 mmHg increase in cumulative BP.

c Effect sizes represent associations per 20 mmHg increase in cumulative BP;.

⁎ Pack year, Cumulative BP=BP_1_*(time_2_-time_1_) + …. + BP_n_*(time_n+1_-time_n_) + BP_n+1_;.

Of seven cohort studies that assessed the association between cumulative DBP and composite cognitive scores, three cohorts[30]^,^ [32,34] found no association; two cohorts[25,30] reported a negative association, while another two cohorts[17] reported a positive association. Among the six cohorts that assessed cumulative DBP and memory function, three cohorts[25,30]^,^ [32] found no association, and two cohorts [17] found a positive association. In the VLS study[30], higher cumulative DBP was associated with an increased decline in immediate recall but not with free recall or delayed recall. Among the three cohorts that assessed cumulative DBP and executive function, the ELSA and HRS cohorts[17] reported a negative association, while CARDIA cohort[25] identified a positive association. For cumulative DBP and orientation function, two cohorts provided data: the ELSA cohort[17] reported a positive association, whereas the HRS cohort[17] found no association. Additionally, higher cumulative DBP was associated with decreased verbal fluency in H70 cohort[30], while no association was observed for mental intactness in CHARLS cohort[32]. (Table 2)

### 3.6 Cumulative BP and neuroimaging markers

Two cohorts reported a positive association between cumulative SBP and WMH volume. One study[16] found that increased cumulative SBP was associated with higher WMH volume, another study[29] reported that increased cumulative SBP was linked to greater progression of WMH volume. (Table 2) For cumulative DBP, one study showed that increased cumulative DBP was associated with higher WMH volume[29]. (Table 2)

### 3.7 Cumulative BP and study outcomes by age groups

Three cohort studies examined the association between cumulative SBP and dementia risk among populations aged ≥60 years. Of these, the ELSA cohort [17] reported higher cumulative SBP was associated with increased risk of dementia, while the HRS and MAS cohorts[17]^,^ [34] found no association. Regarding cognitive function, seven cohorts in populations aged ≥60 years were conducted. Among them, three studies [17,27,32] reported that higher cumulative SBP was associated with poorer cognitive outcomes, whereas another 4 studies[30,17]^,34^observed no association. In contrast, all three studies involving populations aged <60 years[25,32] reported higher cumulative SBP was associated with poorer cognitive function. Additionally, the CARDIA cohort conducted in a population aged <60^16^ reported that increased cumulative SBP was associated with higher WMH volume.

For cumulative DBP, three cohort studies examined its association with dementia risk among populations aged ≥60 years. Of these, the HRS cohort[17] identified higher cumulative DBP was associated with decreased risks of dementia, while the ELSA and MAS cohorts[17]^,^ [34] found no association. Regarding cognitive function, in those aged ≥60, 5 cohorts[30,17,34] observed no association, whereas the VLS cohort[30] reported that higher cumulative DBP associated with poorer cognitive performance. In contrast, the CARDIA cohort[25] conducted in those <60 years reported that higher cumulative DBP was associated with poorer cognitive outcomes.

### 3.8 Cumulative BP and study outcomes by sex

The associations between cumulative SBP and dementia or cognitive decline were consistent across both females and males in six cohorts[17,27,30,32,34]. However, the HRS cohort[17] reported sex-specific differences, identifying higher cumulative SBP as a significant risk factor for dementia and associated with poorer cognitive performance in females, but not in males.

For cumulative DBP, higher levels associated with decreased risks of dementia in both the ELSA and HRS cohorts, and with better cognitive performance in the HRS cohort among both males and females [17]. In the H70 cohort[30], cumulative DBP was not associated with cognitive outcomes in either males or females. In the VLS cohort[30], no association was observed in males, whereas higher cumulative DBP associated with poorer cognition performance was noted in females. In the ELSA cohort[17], higher cumulative DBP associated with better cognitive in males but no association in females.

### 3.9 Cumulative BP and study outcomes by hypertension status

In both hypertensive and non-hypertensive individuals, the ELSA cohorts[17] found that higher cumulative SBP was associated with increased risk of dementia and poorer cognitive performance. In the HRS cohort[17], higher cumulative SBP was associated with increased dementia risk in both groups but was significantly associated with cognitive decline only among non-hypertensive individuals.

For cumulative DBP, the HRS cohort[17] reported higher levels associated with decreased risks of dementia and better cognition performance regardless of hypertension status. In contrast, the ELSA cohort[17] found this protective association only among individuals with hypertension, with association in those without hypertension.

### 3.10 Cumulative BP and study outcomes by antihypertensive medication

Among individuals with or without antihypertensive medication, four cohorts [17,29,32] consistently reported positive associations between higher cumulative SBP and increased risks of dementia, cognitive decline, or greater WMH volume. In the ELSA cohort, higher cumulative SBP was associated with cognitive decline among individuals not using antihypertensive medication, whereas no association was observed among those receiving antihypertensive treatment.

**Fig. 3:**
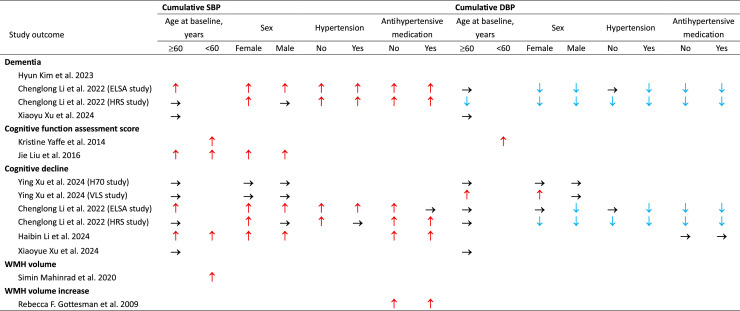
**Summary of reported associations between higher cumulative BP and study outcomes by different subgroups**. **↑**, Increased risk; **→**, NO effect; ↓,Decreased risk. To summarize subgroup findings, we prioritized the reporting of results of MMSE, if MMSE score was unavailable we reported the results from composite score. SBP, systolic blood pressure; DBP, diastolic blood pressure.

For cumulative DBP, higher levels associated with decreased dementia risks and better cognitive performance regardless of antihypertensive medication use in both the ELSA and HRS cohorts[17]. In the CHARLS cohort[17], they found no association with cognition regardless of antihypertensive medication use. (Fig. 3)

### 3.11 Study quality and bias assessment

Overall, all of the included publications were based on high-quality cohort studies [16,17,[23], [24], [25], [26], [27], [28], [29], [30], [31], [32], [33], [34]] (**Supplementary Table S4**). All studies were conducted in general population samples that included both females and males, and all accounted for potential confounding by adjusting for relevant covariates in their analyses. For the studies included in the analysis, three studies were assessed to be at low risk of bias[16,27,31], six studies were assessed as having low to moderate risk [17,29,30]^,^ [32,34], and two studies were rated as having moderate overall risk of bias[17,25]. (**Supplementary Table S5**). The primary sources of bias identified were related to inadequate adjustment for confounding variables, variation in the methods used to measure cumulative BP, and inconsistencies in outcome ascertainment across the studies.

## 4 Discussion

Overall, we included 10 independent cohort studies from 9 publications in our qualitative and quantitative analyses. Meta-analysis showed that higher cumulative SBP was associated with increased dementia risk, whereas cumulative DBP demonstrated no significant association. Regarding cognitive function, five cohorts reported that higher cumulative SBP was associated with poorer cognitive performance, although three cohorts reported non-significant associations. In contrast, the findings for higher cumulative DBP were more mixed, including positive, inverse, and null associations. In terms of neuroimaging outcomes, two studies found that higher cumulative SBP and DBP were associated with increased WMH burden. Sensitivity analyses stratified by age and subgroup analyses indicated that higher cumulative SBP was consistently associated with increased dementia risk and poorer cognitive performance in individuals aged <60 years, whereas an inverse association between higher cumulative DBP and dementia was observed only among those aged ≥60 years. Other subgroup analyses by sex, hypertension status, and use of antihypertensive medication generally showed consistent results across most studies.

Higher cumulative SBP may impair the structural and functional integrity of cerebral vessels[4], supporting its association with increased risk of dementia and cognitive impairment. For cumulative DBP, several studies in this review reported an inverse association with dementia risk and cognitive function. This relationship may be explained by several mechanisms. First, when SBP is elevated but DBP is low, it may indicate increased arterial stiffness and reduced cerebral perfusion, both of which are detrimental to brain health. In contrast, maintaining DBP at an adequate level may help sustain cerebral blood flow, thereby providing neuroprotective effects[38,39]. Additionally, persistently high SBP could lead to left ventricular hypertrophy, which may subsequently lower DBP[40]. Increased left ventricular mass has been associated with poorer cognitive outcomes in older adults[41]. However, the mechanisms underlying these associations are likely complex and interrelated. Given the physiological interplay between SBP and DBP, future studies should evaluate their combined effects rather than examining them separately. This underscores the value of assessing cumulative pulse pressure (PP) and mean arterial pressure (MAP) as complementary metrics. Furthermore, the observed non-linear associations between DBP and dementia may also help to explain these inverse findings. Rather than indicating a true protective effect of higher cumulative DBP, such patterns may reflect J- or U-shaped relationships and survival or selection bias[11,12].

Dementia and cognitive decline are known to be age-related conditions. Previous studies have demonstrated that elevated BP in midlife, rather than in later life, is a stronger predictor of late-life cognitive impairment[42]. Effective BP control during midlife has been recommended as a key preventive strategy against cognitive decline and dementia in older age[43]. In our study, the positive association of cumulative SBP with dementia-related outcomes were more pronounced among middle-aged adults, supporting the hypothesis that BP control during midlife may be particularly important for preventing late-life cognitive impairment and dementia. While the inverse association of higher cumulative DBP was observed only among individuals aged ≥60 years, suggesting that the relationship between cumulative DBP and cognitive outcomes may be influenced by more complex regulatory mechanisms, particularly in older populations and the well-established patterns of relative change in SBP and DBP with ageing such that SBP tends to rise with increasing age whereas DBP rises to around age 60 but then falls representing increasing arterial stiffness[44]. Future work stratifying analyses by age group is essential for understanding how cumulative BP contributes differently to dementia risk across the life course.

This systematic review is the first to synthesize existing evidence on the association between cumulative BP and the subsequent risk of dementia and cognitive decline in adults. These findings underscore the importance of early and sustained SBP management, particularly during midlife, to reduce the long-term risk of cognitive decline and dementia. They also suggest that cumulative SBP and DBP may have differential associations with cognitive outcomes, emphasizing the need to maintain adequate DBP levels in older adults. These observations warrant greater consideration of both age and BP components when developing anti-hypertensive treatment strategies.

All included studies were derived from high-quality longitudinal cohorts, in which the risk of bias was low to moderate. The studies were conducted across six countries spanning North America, Asia, Europe, and Oceania. However, there are limitations. First, there was substantial between-study heterogeneity in the meta-analyses, which reduces the precision and interpretability of the pooled estimates and means that the findings should be interpreted with caution. Second, the age structure of the included cohorts varied widely, and data for the very old (e.g.>80 years) were difficult to extract, limiting our ability to characterise life-course patterns and to generalise the results to the oldest-old. Third, methods for defining dementia and cognitive outcomes varied considerably across the included studies. These differences in outcome ascertainment could affect the sensitivity and specificity of outcome detection, potentially influencing the observed associations. Fourth, dementia subtypes (e.g. Alzheimer’s disease and vascular dementia) were not consistently reported across cohorts, which constrained us from conducting separate meta-analyses by dementia subtype and limits the extent to which our findings can be interpreted in relation to specific dementia aetiologies. While, mixed dementia aetiology is common in late life, and clinical dementia subtypes often do not reflect a single underlying pathology because dementia pathology frequently overlaps[45]. Fifth, among the studies that met our eligibility criteria for cumulative BP, only two reported WMH outcomes, none reported hippocampal volume in relation to cumulative BP exposure, and cerebrospinal fluid or blood-based Alzheimer’s disease biomarkers (e.g. Aβ and tau measures) were not considered as outcomes in this review. These limitations restrict our ability to comment on intermediate pathophysiological pathways. Furthermore, heterogeneity in covariate adjustment between studies and the potential impact of time-varying confounding may have biased the observed associations.

To strengthen the evidence and guide tailored recommendations for specific populations, future research should aim to enhance both the methodological rigor and scope of studies in this field. Specifically, future investigations should: (1) standardize the assessment of cumulative BP to enhance comparability across studies, with AUC currently being the most commonly used method; (2) utilize well-designed prospective cohort studies with BP measurements over long follow-up periods to accurately capture long-term exposure; (3) account for time-dependent factors such as antihypertensive medication use, comorbidities, and lifestyle changes that may influence both BP and cognitive outcomes; (4) ensure clear and robust diagnostic criteria for dementia and distinguish between dementia subtypes (e.g., pure or predominant Alzheimer's disease or vascular dementia) to explore potential subtype-specific associations; (5) use standardized and comprehensive cognitive assessments (e.g., MMSE), and also to detect domain-specific impairments (e.g., memory, executive function, attention); (6) include diverse populations across age, sex, ethnicity, geographic regions, and disease histories to improve the generalizability of findings; and (7) assess the associations of cumulative PP and MAP with dementia-related outcomes; (8) derive both cumulative BP and BP variability measures to assess their independent and potentially interacting contributions to dementia-related outcomes.

## 5 Conclusion

Higher cumulative SBP was associated with increased risks of dementia and poorer cognitive performance. In contrast, the relationship between cumulative DBP and these outcomes appears to be mixed. Stratified analyses by age group, as well as investigations into the combined effect of cumulative SBP and DBP, are essential to clarify these associations. Further research using well-designed prospective cohort studies is needed to better define these relationships and inform clinical strategies.

## Funding

None

## Declaration of generative AI and AI-assisted technologies in the writing process

None

## Ethical statement

Consent was not necessary.

## Data statement

Data was extracted and analyzed using meta-analytic techniques.

## CRediT authorship contribution statement

**Ruirui Wang:** Writing – original draft, Formal analysis, Conceptualization. **Yijie Gao:** Formal analysis, Conceptualization. **Nicole Ee:** Formal analysis, Conceptualization. **Fope Akinyede:** Formal analysis. **Xiaoyue Xu:** Formal analysis, Data curation. **Linan Chen:** Formal analysis. **Shangzhi Xiong:** Formal analysis. **Xiaoying Chen:** Supervision, Data curation. **Craig S. Anderson:** Writing – review & editing, Supervision. **Katie Harris:** Writing – review & editing, Supervision. **Ruth Peters:** Writing – review & editing, Supervision, Data curation, Conceptualization.

## Declaration of competing interest

The authors declare the following financial interests/personal relationships which may be considered as potential competing interests:

Yijie Gao reports financial support was provided by University of New South Wales. If there are other authors, they declare that they have no known competing financial interests or personal relationships that could have appeared to influence the work reported in this paper.

## Appendix Supplementary materials

Image, application 1
