# Supplementary material for: Cumulative blood pressure and risk of dementia and cognitive decline: a systematic review and meta-analysis
Source: J Prev Alzheimers Dis. 2026 Feb 7;13(4):100500. doi: 10.1016/j.tjpad.2026.100500 (PMC12907844; doi:10.1016/j.tjpad.2026.100500)
Supplement: Supplementary file 1 [file mmc1.docx]

**Supplementary material**

**Table S1. Search strategy.**

| **PubMed** | | |
| --- | --- | --- |
| Search number | Query | Results |
| 1 | ("Cumulative" OR "Burden" OR "Lifetime" OR "Time-dependent" OR "Time-weighted") AND ("blood pressure" OR "BP" OR "pressure" OR "MAP" OR "Hypertension exposure") | [39,860](https://pubmed.ncbi.nlm.nih.gov/?term=%28%22Cumulative%22+OR+%22Burden%22+OR+%22Lifetime%22+OR+%22Time-dependent%22+OR+%22Time-weighted%22%29+AND+%28%22blood+pressure%22+OR+%22BP%22+OR+%22pressure%22+OR+%22MAP%22+OR+%22Hypertension+exposure%22%29&ac=no&sort=relevance) |
| 2 | Cognit*[tiab] OR dement*[tiab] OR neurocogn*[tiab] OR Alzheimer*[tiab] OR Neurodegen*[tiab] OR ((mental[tiab] OR memory[tiab]) AND (Disorders[tiab] OR decline[tiab] OR impairment[tiab] OR Deterioration[tiab] OR syndromes[tiab] OR Dysfunction[tiab])) | [1,060,214](https://pubmed.ncbi.nlm.nih.gov/?term=Cognit%2A%5Btiab%5D+OR+dement%2A%5Btiab%5D+OR+neurocogn%2A%5Btiab%5D+OR+Alzheimer%2A%5Btiab%5D+OR+Neurodegen%2A%5Btiab%5D+OR+%28%28mental%5Btiab%5D+OR+memory%5Btiab%5D%29+AND+%28Disorders%5Btiab%5D+OR+decline%5Btiab%5D+OR+impairment%5Btiab%5D+OR+Deterioration%5Btiab%5D+OR+syndromes%5Btiab%5D+OR+Dysfunction%5Btiab%5D%29%29&ac=no&sort=relevance) |
| 3 | "Cognition"[Mesh:NoExp] OR "Cognitive Dysfunction"[Mesh:NoExp] OR "Cognition Disorders"[Mesh:NoExp] OR "Neurocognitive Disorders"[Mesh:NoExp] OR "Dementia"[Mesh] OR "Memory"[Mesh:NoExp] | [479,821](https://pubmed.ncbi.nlm.nih.gov/?term=%22Cognition%22%5BMesh%3ANoExp%5D+OR+%22Cognitive+Dysfunction%22%5BMesh%3ANoExp%5D+OR+%22Cognition+Disorders%22%5BMesh%3ANoExp%5D+OR+%22Neurocognitive+Disorders%22%5BMesh%3ANoExp%5D+OR+%22Dementia%22%5BMesh%5D+OR+%22Memory%22%5BMesh%3ANoExp%5D&ac=no&sort=relevance) |
| 4 | #2 OR #3 | [1,176,416](https://pubmed.ncbi.nlm.nih.gov/?term=%232+OR+%233&ac=no&sort=relevance) |
| 5 | #1 AND #4 | [1,618](https://pubmed.ncbi.nlm.nih.gov/?term=%231+AND+%234&ac=no&sort=relevance) |
| **Embase (Ovid)** | | |
| Search number | Query | Results |
| 1 | ((Cumulative or Burden or Lifetime or Time-dependent or Time-weighted) and ("blood pressure" or BP or pressure or MAP or "Hypertension exposure")).mp. | 56,585 |
| 2 | (Cognit* or dement* or neurocogn* or Alzheimer* or Neurodegen* or ((mental or memory) and (Disorders or decline or impairment or Deterioration or syndromes or Dysfunction))).ab,kf,ti. | 1,423,360 |
| 3 | Cognition/ or "Cognitive Dysfunction"/ or "Cognition Disorders"/ or "Neurocognitive Disorders"/ or exp Dementia/ or Memory/ | 1,040,146 |
| 4 | 2 or 3 | 1,715,233 |
| 5 | 1 and 4 | 3,232 |
| **Web of Science** | | |
| Search number | Query | Results |
| 1 | TS=((Cumulative or Burden or Lifetime or Time-dependent or Time-weighted) and ("blood pressure" or BP or pressure or MAP or "Hypertension exposure")) | [94,785](https://www-webofscience-com.wwwproxy1.library.unsw.edu.au/wos/woscc/summary/f8e6c3b9-178b-449f-af23-621385272b39-0143d78dc5/relevance/1) |
| 2 | TS=(Cognit* or dement* or neurocogn* or Alzheimer* or Neurodegen* or (((mental OR memory) NEAR/2 (Disorders OR decline OR impairment OR Deterioration OR syndromes OR Dysfunction)))) | [1,591,398](https://www-webofscience-com.wwwproxy1.library.unsw.edu.au/wos/woscc/summary/6110fe49-4e3d-4c9c-9e47-c90e7fabfe84-0143d7950e/relevance/1) |
| 3 | #1 AND #2 | [3,146](https://www-webofscience-com.wwwproxy1.library.unsw.edu.au/wos/woscc/summary/28ec92e1-31af-42bd-8b5f-8424c15896a1-0143d79691/relevance/1) |
| **Cochrane library** | | |
| Search number | Query | Results |
| 1 | (Cumulative OR Burden OR Lifetime OR Time-dependent OR Time-weighted ) AND ("blood pressure" OR BP OR pressure OR MAP OR "Hypertension exposure" ) | 7,893 |
| 2 | Cognit*:ti,ab OR dement*:ti,ab OR neurocogn*:ti,ab OR Alzheimer*:ti,ab OR Neurodegen*:ti,ab OR ((mental:ti,ab OR memory:ti,ab) AND (Disorders:ti,ab OR decline:ti,ab OR impairment:ti,ab OR Deterioration:ti,ab OR syndromes:ti,ab OR Dysfunction:ti,ab)) | 139,797 |
| 3 | [mh ^Cognition] OR [mh ^"Cognitive Dysfunction"] OR [mh ^"Cognition Disorders"] OR [mh ^"Neurocognitive Disorders"] OR [mh Dementia] OR [mh ^Memory] | 29,535 |
| 4 | #2 OR #3 | 144,174 |
| 5 | #1 AND #4 | 760 |
| **CNKI** | | |
| Search number | Query | Results |
| 1 | (篇关摘：血压 + 收缩压 + 舒张压 + 高血压) AND (篇关摘：累积 + 负担 + 时间依赖 + 时间加权 + 终身) | 4491 |
| 2 | (篇关摘：痴呆 + 阿尔茨海默 + 认知 + 脑白质 + 淀粉样蛋白) | 578,915 |
| 3 | #1 AND #2 | 204 |

**Table S2.** **Reasons for Exclusion**

| **Title** | **Exclusion reason** |
| --- | --- |
| Associations Between Vascular Risk Factor Levels and Cognitive Decline Among Stroke Survivors.[1] | Wrong exposure |
| Year-by-Year Blood Pressure Variability From Midlife to Death and Lifetime Dementia Risk.[2] | Wrong exposure |
| Midlife Consequences of Cumulative Blood Pressure Exposure: Importance of a Lifespan Approach.[3] | Review or editorial or other; |
| Longitudinal trajectories of Alzheimer's disease CSF biomarkers and blood pressure in cognitively healthy subjects.[4] | Wrong exposure |
| Mediation Effect of Left Ventricular Geometric Adaptation to Lifetime Blood Pressure on Cognitive Function in Middle-Age: The Heart-Brain Connection (Partially) Explained.[5] | Review or editorial or other |
| Beyond Hypertension: Examining Variable Blood Pressure's Role in Cognition and Brain Structure.[6] | Wrong exposure |
| Associations of hypertension burden on subsequent dementia: a population-based cohort study.[7] | Wrong exposure |
| Association Between Blood Pressure and Later-Life Cognition Among Black and White Individuals.[8] | Wrong effect measurements |
| Pathological Continuum From the Rise in Pulse Pressure to Impaired Neurovascular Coupling and Cognitive Decline.[9] | Review or editorial or other |
| Presymptomatic Profiles of Cognitive Impairment with Prior Mobility Impairment.[10] | Wrong exposure |
| Antemortem Visit-To-Visit Blood Pressure Variability Predicts Cerebrovascular Lesion Burden in Autopsy-Confirmed Alzheimer's Disease.[11] | Wrong outcomes |
| Associations of Blood Pressure with Functional and Cognitive Changes in Patients with Alzheimer's Disease.[12] | Wrong exposure |
| Blood Pressure and Later-Life Cognition in Hispanic and White Adults (BP-COG): A Pooled Cohort Analysis of ARIC, CARDIA, CHS, FOS, MESA, and NOMAS.[13] | Wrong effect measurement |
| Blood pressure and cognitive function across the eighth decade: a prospective study of the Lothian Birth Cohort of 1936.[14] | Wrong exposure |
| Self-Reported Late-Life Hypertension Is Associated with a Healthy Cognitive Status and Reduced Alzheimer's Disease Pathology Burden.[15] | Wrong exposure |
| Distinct effects of blood pressure parameters on Alzheimer's and vascular markers in 1,952 Asian individuals without dementia.[16] | Wrong exposure |
| Observational Study on Cognitive function And systolic blood pressure Reduction (OSCAR): preliminary analysis of 6-month data from > 10,000 patients and review of the literature.[17] | Wrong exposure |
| Cardiovascular Risk Factors From Childhood and Midlife Cognitive Performance: The Young Finns Study.[18] | Wrong patient population |
| [Prevention of dementia (including Alzheimer's disease)].[19] | Review or editorial or other |
| The effects of an exercise and lifestyle intervention program on cardiovascular, metabolic factors and cognitive performance in middle-aged adults with type II diabetes: a pilot study.[20] | Wrong exposure |
| Risk factors for dementia in the ninth decade of life and beyond: a study of the Lothian birth cohort 1921.[21] | Wrong patient population |
| The potential impact of optimal blood pressure treatment intensity to reduce disparities in dementia between Black and White individuals.[22] | Wrong exposure |
| Optimizing Blood Pressure Components for a Healthy Brain: The Holy Grail in Blood Pressure Management.[23] | Review or editorial or other |
| Right care, first time: a highly personalised and measurement-based care model to manage youth mental health.[24] | Wrong exposure |
| Systolic blood pressure variability in late-life predicts cognitive trajectory and risk of Alzheimer's disease.[25] | Wrong exposure |
| Blood Pressure Trajectories and Amyloid- and Tau-PET Burden in Cognitively Healthy Adults: The Framingham Heart Study[26] | Wrong outcomes |
| Rethinking Blood Pressure Treatment and Dementia Risk in Older Adults: Is Adherence the Holy Grail?[27] | Review or editorial or other |
| The quid pro quo of blood pressure control and dementia[28] | Review or editorial or other |
| Independent Associations of Education, Intelligence, and Cognition with Hypertension and the Mediating Effects of Cardiometabolic Risk Factors: A Mendelian Randomization Study[29] | Wrong study design |
| CUMULATIVE BLOOD PRESSURE LOAD AND INCIDENT DEMENTIA, COGNITIVE FUNCTION, AND ALL-CAUSE AND CARDIOVASCULAR DEATHS IN ADULTS AGED 70 YEARS AND OLDER[30] | No full text |
| ASSOCIATIONS OF DIFFERENT BLOOD PRESSURE (BP) PARAMETERS WITH COGNITIVE DECLINE AND DEMENTIA IN PATIENTS WITH MILD COGNITIVE IMPAIRMENT (MCI) AND DIABETES[31] | No full text |
| Cumulative Blood Pressure Load and Incident Dementia, Cognitive Function, and All-cause and Cardiovascular Deaths in Older Adults[32] | No full text |
| Associations of beta-Amyloid and Vascular Burden with Rates of Neurodegeneration in Cognitively Normal Members of the 1946 British Birth Cohort[33] | Wrong exposure |
| Preserving Cognition, Preventing Dementia[34] | Review or editorial or other |
| Effect of past and concurrent elevated blood pressure on white matter hyperintensities: An analysis of the uk biobank cohort[35] | No full text |
| Risk of vascular disease and cognitive function in very old adults[36] | Review or editorial or other |
| Blood pressure over the life course and later-life cognition in blacks, hispanics, and whites (BP-COG): Pooled cohort analysis of ARIC, CARDIA, CHS, FOS, and NOMAS[37] | Wrong effect measurement |
| HIGHER CUMULATIVE EXPOSURE TO BLOOD PRESSURE DURING YOUNG ADULTHOOD THROUGH MIDLIFE PREDICTS REDUCED LOCAL FUNCTIONAL HIPPOCAMPAL CONNECTIVITY AT REST IN MIDLIFE[38] | Wrong outcomes |
| Cumulative exposure to elevated blood pressure throughout adulthood and late-life dementia risk[39] | No full text |
| Racial/ethnic differences in the association of systolic blood pressure across midlife and late life on cognitive function: The multi-ethnic study of atherosclerosis[40] | No full text |
| Prevention of dementia by intensive vascular care (prediva): A cluster randomized trial[41] | Wrong exposure |
| Vascular risk factors and cognitive function in midlife women: The study of women's health across the nation (SWAN)[42] | No full text |
| Low cardiac index is associated with incident dementia and Alzheimer's disease: The framingham heart study[43] | Wrong exposure |
| Nocturnal blood pressure in young adults and cognitive function in midlife: The CARDIA study[44] | No full text |
| Long-term blood pressure variability though young adulthood and cognitive function in midlife; the coronary artery risk development in young adults (cardia)study[45] | Wrong exposure |
| The importance of risk factor modification over the lifecourse[46] | No full text |
| Risk factors for blood transfusion in patients undergoing hip fracture surgery[47] | Wrong exposure |
| Cardiovascular risk factors for cognitive function: Effects from early adulthood to mid-life[48] | No full text |
| A study of blood pressure and change in cognitive function in elderly mexican Americans[49] | No full text |
| Blood pressure, treatment and dementia risk in older Mexican Americans[50] | No full text |
| Impact of gender and blood pressure on post-stroke cognitive decline among older latinos[51] | No full text |
| Blood pressure and white matter disease progression in a biethnic cohort[52] | No full text |
| Prevention of dementia by intensive vascular care: Prediva[53] | Wrong effect measurement |
| Repetitive hypotensive episodes - Their contribution to vascular cognitive impairment[54] | Wrong exposure |
| Blood Pressure and Hispanic/Latino Cognitive Function: Hispanic Community Health Study/Study of Latinos Results[55] | Wrong exposure |
| Association between blood pressure control status, visit-to-visit blood pressure variability, and cognitive function in elderly Chinese: A nationwide study[56] | Wrong exposure |
| Long-Term Blood Pressure Variability and Risk of Cognitive Decline and Dementia Among Older Adults[57] | Wrong exposure |
| Longitudinal associations of cardiovascular health and vascular events with incident dementia[58] | Wrong exposure |
| Cumulative Blood Pressure Levels predictive for Dementia Risk[59] | No full text |
| Relative importance of potential risk factors for dementia in patients with hypertension[60] | Wrong exposure |
| The 65 trial[61] | No full text |
| 血压、动脉僵硬度与认知的关系:基于2个人群(儿童和成人)的队列研究[62] | Wrong exposure |
| 血压变异性与认知功能障碍相关性的研究进展[63] | Review or editorial or other |
| 高血压合并老年痴呆患者动态血压分析[64] | Wrong exposure |
| 帕金森病患者血压变化与认知功能的研究进展[65] | Review or editorial or other |
| 高血压对认知功能的影响[66] | Review or editorial or other |
| 老年高血压与认知功能的研究进展[67] | Review or editorial or other |

**Table S3. Odds ratios (95% CI) for the associations between per SD increase in cumulative BP and risk of dementia after excluding younger cohort.**

| Studies | Sample size | Cumulative BP duration | Cumulative BP measurements | Cumulative SBP | Cumulative DBP |
| --- | --- | --- | --- | --- | --- |
|  |  |  |  | OR (95% CI) | OR (95% CI) |
| Chenglong Li et al. 2022 (ELSA study) | N=7566 | 5-10 years | AUC | 1.28 (1.17 to 1.41) | 0.81 (0.74 to 0.88) |
| Chenglong Li et al. 2022 (HRS study) | N=9294 | <5 years | AUC | 1.16 (1.10 to 1.23) | 0.81 (0.77 to 0.86) |
| Xiaoyue Xu et al. 2024 | N=1037 | 5-10 years | AUC | 0.93 (0.79 to 1.11) | 0.89 (0.76 to 1.03) |
| Overall |  |  |  | 1.13 (0.95 to 1.34) | 0.82 (0.78 to 0.85) |

AUC, area under the curve (mmHg*time); SBP, systolic blood pressure; DBP, diastolic blood pressure.

Cumulative SBP: P for heterogeneity=0.005, I^2^=89.07%, 𝜏^2^=0.019.

Cumulative DBP: P for heterogeneity=0.510, I^2^=0.05%, 𝜏^2^=0.000.

**Table S4. Quality assessment**

|  |  | **Simin Mahinrad et al. 2020** | **Lisanne M. Jenkinsa et al. 2021** | **Christina S. Dintica1 et al. 2022** | **Kristine Yaffe et al. 2014** | **Lisanne M. Jenkinsa et al. 2020** | **Jie Liu et al. 2016** | **Huijing Shi et al. 2023** | **Rebecca F. Gottesman et al. 2009** | **Ying Xu et al. 2024 (H70 study)** | **Ying Xu et al. 2024 (VLS study)** | **Hyun Kim et al. 2023** | **Chenglong Li et al. 2022 (ELSA study)** | **Chenglong Li et al. 2022 (HRS study)** | **Haibin Li et al. 2024** | **Lili Luo et al. 2024** | **Xiaoyue Xu et al. 2024** |
| --- | --- | --- | --- | --- | --- | --- | --- | --- | --- | --- | --- | --- | --- | --- | --- | --- | --- |
| **Selection** |  |  |  |  |  |  |  |  |  |  |  |  |  |  |  |  |  |
| **1)** | **Representativeness of the exposed cohort** |  |  |  |  |  |  |  |  |  |  |  |  |  |  |  |  |
|  | a) Truly representative (one star) | ☆ |  |  | ☆ |  |  |  | ☆ |  |  | ☆ |  |  |  |  | ☆ |
|  | b) Somewhat representative (one star) |  |  |  |  |  | ☆ | ☆ |  | ☆ | ☆ |  | ☆ | ☆ | ☆ | ☆ |  |
|  | c) Selected group |  | X | X |  | X |  |  |  |  |  |  |  |  |  |  |  |
|  | d) No description of the derivation of the cohort |  |  |  |  |  |  |  |  |  |  |  |  |  |  |  |  |
| **2)** | **Selection of the non-exposed cohort** |  |  |  |  |  |  |  |  |  |  |  |  |  |  |  |  |
|  | a) Drawn from the same community as the exposed cohort (one star) | ☆ | ☆ | ☆ | ☆ | ☆ | ☆ | ☆ | ☆ | ☆ | ☆ | ☆ | ☆ | ☆ | ☆ | ☆ | ☆ |
|  | b) Drawn from a different source |  |  |  |  |  |  |  |  |  |  |  |  |  |  |  |  |
|  | c) No description of the derivation of the non exposed cohort |  |  |  |  |  |  |  |  |  |  |  |  |  |  |  |  |
| **3)** | **Ascertainment of exposure** |  |  |  |  |  |  |  |  |  |  |  |  |  |  |  |  |
|  | a) Secure record (e.g., surgical record) (one star) | ☆ | ☆ | ☆ | ☆ | ☆ | ☆ | ☆ | ☆ | ☆ | ☆ | ☆ | ☆ | ☆ | ☆ | ☆ | ☆ |
|  | b) Structured interview (one star) |  |  |  |  |  |  |  |  |  |  |  |  |  |  |  |  |
|  | c) Written self report |  |  |  |  |  |  |  |  |  |  |  |  |  |  |  |  |
|  | d) No description |  |  |  |  |  |  |  |  |  |  |  |  |  |  |  |  |
|  | e) Other |  |  |  |  |  |  |  |  |  |  |  |  |  |  |  |  |
| **4)** | **Demonstration that outcome of interest was not present at start of study** |  |  |  |  |  |  |  |  |  |  |  |  |  |  |  |  |
|  | a) Yes (one star) | ☆ | ☆ | ☆ | ☆ | ☆ |  | ☆ | ☆ | ☆ | ☆ | ☆ | ☆ | ☆ |  | ☆ |  |
|  | b) No |  |  |  |  |  | X |  |  |  |  |  |  |  | X |  | X |
| **Comparability** |  |  |  |  |  |  |  |  |  |  |  |  |  |  |  |  |  |
| **1)** | **Comparability of cohorts on the basis of the design or analysis controlled for confounders** |  |  |  |  |  |  |  |  |  |  |  |  |  |  |  |  |
|  | a) The study controls for age, sex and marital status (one star) | ☆ | ☆ | ☆ | ☆ | ☆ | ☆ | ☆ | ☆ | ☆ | ☆ | ☆ | ☆ | ☆ | ☆ | ☆ | ☆ |
|  | b) Study controls for other factors education, race (one star) | ☆ | ☆ | ☆ | ☆ | ☆ | ☆ | ☆ | ☆ | ☆ | ☆ | ☆ | ☆ | ☆ | ☆ | ☆ | ☆ |
|  | c) Cohorts are not comparable on the basis of the design or analysis controlled for confounders |  |  |  |  |  |  |  |  |  |  |  |  |  |  |  |  |
| **Outcome** |  |  |  |  |  |  |  |  |  |  |  |  |  |  |  |  |  |
| **1)** | **Assessment of outcome** |  |  |  |  |  |  |  |  |  |  |  |  |  |  |  |  |
|  | a) Independent blind assessment (one star) | ☆ | ☆ | ☆ | ☆ | ☆ | ☆ | ☆ | ☆ | ☆ | ☆ | ☆ | ☆ | ☆ | ☆ | ☆ | ☆ |
|  | b) Record linkage (one star) |  |  |  |  |  |  |  |  |  |  |  |  |  |  |  |  |
|  | c) Self report |  |  |  |  |  |  |  |  |  |  |  |  |  |  |  |  |
|  | d) No description |  |  |  |  |  |  |  |  |  |  |  |  |  |  |  |  |
|  | e) Other |  |  |  |  |  |  |  |  |  |  |  |  |  |  |  |  |
| **2)** | **Was follow-up long enough for outcomes to occur** |  |  |  |  |  |  |  |  |  |  |  |  |  |  |  |  |
|  | a) Yes (one star) | ☆ | ☆ | ☆ | ☆ | ☆ |  | ☆ | ☆ | ☆ | ☆ | ☆ | ☆ | ☆ |  | ☆ |  |
|  | b) No |  |  |  |  |  | X |  |  |  |  |  |  |  | X |  | X |
|  | Indicate the median duration of follow-up and a brief rationale for the assessment above:____________________ |  |  |  |  |  |  |  |  |  |  |  |  |  |  |  |  |
| **3)** | **Adequacy of follow-up of cohorts** |  |  |  |  |  |  |  |  |  |  |  |  |  |  |  |  |
|  | a) Complete follow up- all subject accounted for (one star) |  |  |  |  |  |  |  |  |  |  |  |  |  |  |  |  |
|  | b) Subjects lost to follow up unlikely to introduce bias- number lost less than or equal to 20% or description of those lost suggested no different from those followed. (one star) | ☆ | ☆ | ☆ | ☆ | ☆ | ☆ | ☆ | ☆ |  |  | ☆ | ☆ | ☆ | ☆ |  |  |
|  | c) Follow up rate less than 80% and no description of those lost |  |  |  |  |  |  |  |  | X | X |  |  |  |  |  |  |
|  | d) No statement |  |  |  |  |  |  |  |  |  |  |  |  |  |  | X | X |
|  |  |  |  |  |  |  |  |  |  |  |  |  |  |  |  |  |  |
| **Quality** | Good quality: 3 or 4 stars in selection domain AND 1 or 2 stars in comparability domain AND 2 or 3 stars in outcome/exposure domain.  Fair quality: 2 stars in selection domain AND 1 or 2 stars in comparability domain AND 2 or 3 stars in outcome/exposure domain.  Poor quality: 0 or 1 star in selection domain OR 0 stars in comparability domain OR 0 or 1 stars in outcome/exposure domain. | Good | Good | Good | Good | Good | Good | Good | Good | Good | Good | Good | Good | Good | Good | Good | Good |

**Table S5. Risk of bias assessment**

| **Study ID** | **Confounding** | **Selection of Participants** | **Measurement of exposure** | **Post-exposure interventions** | **Missing Data** | **Measurement of Outcomes** | **Selective reporting** | **Overall Risk of Bias** |
| --- | --- | --- | --- | --- | --- | --- | --- | --- |
| Simin Mahinrad et al. 2020 | Low | Low | Low | Low | Low | Low | Low | Low |
| Lisanne M. Jenkinsa et al. 2021 | Moderate (Age, sex, race, education, and site) | Low | Low | Low | Low | Moderate (only stroop score) | Low | Moderate |
| Christina S. Dintica1 et al. 2022 | Moderate (Age, sex, education, race) | Low | Moderate (AUC/time) | Low | Low | Low | Low | Moderate |
| Kristine Yaffe et al. 2014 | Moderate (Age, sex, education, race) | Low | Low | Low | Moderate (not report number for each outcome) | Low | Low | Moderate |
| Lisanne M. Jenkinsa et al. 2020 | Moderate (age, sex, race, and intracranial volume) | Low | Low | Low | Low | Low | Low | Low-moderate |
| Jie Liu et al. 2016 | Low | Low | Low | Low | Low | Low | Low | Low |
| Huijing Shi et al. 2023 | Low | Low | Moderate (BP measurement methods not consistent) | Low | Low | Low | Moderate (no continuous score results) | Moderate |
| Rebecca F. Gottesman et al. 2009 | Low | Low | Moderate (AUC/time) | Low | Low | Low | Low | Low-moderate |
| Ying Xu et al. 2024 (H70 study) | Low | Low | Moderate (not AUC) | Low | Low | Low | Low | Low-moderate |
| Ying Xu et al. 2024 (VLS study) | Low | Low | Moderate (not AUC) | Low | Low | Low | Low | Low-moderate |
| Hyun Kim et al. 2023 | Low | Low | Low | Low | Low | Low | Low | Low |
| Chenglong Li et al. 2022 (ELSA study) | Low | Low | Low | Low | Low | Moderate (not clinical diagnosed dementia) | Low | Low-moderate |
| Chenglong Li et al. 2022 (HRS study) | Low | Low | Moderate (duration<5 years) | Low | Low | Moderate (not clinical diagnosed dementia) | Low | Moderate |
| Haibin Li et al. 2024 | Low | Low | Moderate (duration<5 years) | Low | Low | Low | Low | Low-moderate |
| Lili Luo et al. 2024 | Low | Low | Low | Low | Low | Low | Moderate (no continuous score results) | Low-moderate |
| Xiaoyue Xu et al. 2024 | Low | Low | Low | Low | Low | Low | Moderate (only the results of composite tests score | Low-moderate |

**References**

1. Levine, D.A., et al., *Associations Between Vascular Risk Factor Levels and Cognitive Decline Among Stroke Survivors.* JAMA Netw Open, 2023. **6**(5): p. e2313879.

2. den Brok, M., et al., *Year-by-Year Blood Pressure Variability From Midlife to Death and Lifetime Dementia Risk.* JAMA Netw Open, 2023. **6**(10): p. e2340249.

3. Jefferson, A.L., *Midlife Consequences of Cumulative Blood Pressure Exposure: Importance of a Lifespan Approach.* Circulation, 2020. **141**(9): p. 725-727.

4. Biskaduros, A., et al., *Longitudinal trajectories of Alzheimer's disease CSF biomarkers and blood pressure in cognitively healthy subjects.* Alzheimers Dement, 2024. **20**(7): p. 4389-4400.

5. Bella, J.N., *Mediation Effect of Left Ventricular Geometric Adaptation to Lifetime Blood Pressure on Cognitive Function in Middle-Age: The Heart-Brain Connection (Partially) Explained.* Circ Cardiovasc Imaging, 2020. **13**(8): p. e011325.

6. Morrison, C., et al., *Beyond Hypertension: Examining Variable Blood Pressure's Role in Cognition and Brain Structure.* J Gerontol B Psychol Sci Soc Sci, 2024. **79**(9).

7. Jung, H., et al., *Associations of hypertension burden on subsequent dementia: a population-based cohort study.* Sci Rep, 2021. **11**(1): p. 12291.

8. Levine, D.A., et al., *Association Between Blood Pressure and Later-Life Cognition Among Black and White Individuals.* JAMA Neurol, 2020. **77**(7): p. 810-819.

9. de Montgolfier, O., N. Thorin-Trescases, and E. Thorin, *Pathological Continuum From the Rise in Pulse Pressure to Impaired Neurovascular Coupling and Cognitive Decline.* Am J Hypertens, 2020. **33**(5): p. 375-390.

10. Tian, Q., et al., *Presymptomatic Profiles of Cognitive Impairment with Prior Mobility Impairment.* J Am Med Dir Assoc, 2024. **25**(3): p. 480-487.e2.

11. Sible, I.J., et al., *Antemortem Visit-To-Visit Blood Pressure Variability Predicts Cerebrovascular Lesion Burden in Autopsy-Confirmed Alzheimer's Disease.* J Alzheimers Dis, 2021. **83**(1): p. 65-75.

12. de Oliveira, F.F., et al., *Associations of Blood Pressure with Functional and Cognitive Changes in Patients with Alzheimer's Disease.* Dement Geriatr Cogn Disord, 2016. **41**(5-6): p. 314-23.

13. Levine, D.A., et al., *Blood Pressure and Later-Life Cognition in Hispanic and White Adults (BP-COG): A Pooled Cohort Analysis of ARIC, CARDIA, CHS, FOS, MESA, and NOMAS.* J Alzheimers Dis, 2022. **89**(3): p. 1103-1117.

14. Altschul, D., J. Starr, and I. Deary, *Blood pressure and cognitive function across the eighth decade: a prospective study of the Lothian Birth Cohort of 1936.* BMJ Open, 2020. **10**(7): p. e033990.

15. Robinson, A.C., et al., *Self-Reported Late-Life Hypertension Is Associated with a Healthy Cognitive Status and Reduced Alzheimer's Disease Pathology Burden.* J Alzheimers Dis, 2024. **98**(4): p. 1457-1466.

16. Lee, S., et al., *Distinct effects of blood pressure parameters on Alzheimer's and vascular markers in 1,952 Asian individuals without dementia.* Alzheimers Res Ther, 2024. **16**(1): p. 125.

17. Shlyakhto, E., *Observational Study on Cognitive function And systolic blood pressure Reduction (OSCAR): preliminary analysis of 6-month data from > 10,000 patients and review of the literature.* Curr Med Res Opin, 2007. **23 Suppl 5**: p. S13-8.

18. Rovio, S.P., et al., *Cardiovascular Risk Factors From Childhood and Midlife Cognitive Performance: The Young Finns Study.* J Am Coll Cardiol, 2017. **69**(18): p. 2279-2289.

19. Kornhuber, H.H., *[Prevention of dementia (including Alzheimer's disease)].* Gesundheitswesen, 2004. **66**(5): p. 346-51.

20. Fiocco, A.J., et al., *The effects of an exercise and lifestyle intervention program on cardiovascular, metabolic factors and cognitive performance in middle-aged adults with type II diabetes: a pilot study.* Can J Diabetes, 2013. **37**(4): p. 214-219.

21. Sibbett, R.A., et al., *Risk factors for dementia in the ninth decade of life and beyond: a study of the Lothian birth cohort 1921.* BMC Psychiatry, 2017. **17**(1): p. 205.

22. Levine, D.A., et al., *The potential impact of optimal blood pressure treatment intensity to reduce disparities in dementia between Black and White individuals.* J Alzheimers Dis, 2025: p. 13872877241302506.

23. Fernández-Jiménez, R. and C. Real, *Optimizing Blood Pressure Components for a Healthy Brain: The Holy Grail in Blood Pressure Management.* J Am Coll Cardiol, 2022. **79**(14): p. 1336-1339.

24. Hickie, I.B., et al., *Right care, first time: a highly personalised and measurement-based care model to manage youth mental health.* Med J Aust, 2019. **211 Suppl 9**: p. S3-S46.

25. Li, X.L., et al., *Systolic blood pressure variability in late-life predicts cognitive trajectory and risk of Alzheimer's disease.* Front Aging Neurosci, 2024. **16**: p. 1448034.

26. Mulligan, M., et al., *Blood Pressure Trajectories and Amyloid- and Tau-PET Burden in Cognitively Healthy Adults: The Framingham Heart Study.* Neurology, 2024. **102**(17 Supplement 1).

27. Wright, C.B. and M. Egle, *Rethinking Blood Pressure Treatment and Dementia Risk in Older Adults: Is Adherence the Holy Grail?* Journal of the American College of Cardiology, 2024. **83**(13): p. 1204EP-1206.

28. Ryan, J. and M.E. Ernst, *The quid pro quo of blood pressure control and dementia.* The Lancet Healthy Longevity, 2023. **4**(9): p. e444EP-e445.

29. Wang, Y., et al., *Independent Associations of Education, Intelligence, and Cognition with Hypertension and the Mediating Effects of Cardiometabolic Risk Factors: A Mendelian Randomization Study.* Hypertension, 2023. **80**(1): p. 192EP-203.

30. Xu, X., et al., *CUMULATIVE BLOOD PRESSURE LOAD AND INCIDENT DEMENTIA, COGNITIVE FUNCTION, AND ALL-CAUSE AND CARDIOVASCULAR DEATHS IN ADULTS AGED 70 YEARS AND OLDER.* Journal of Hypertension, 2023. **41**(Supplement 3): p. e26EP-e27.

31. Shajahan, S., et al., *ASSOCIATIONS OF DIFFERENT BLOOD PRESSURE (BP) PARAMETERS WITH COGNITIVE DECLINE AND DEMENTIA IN PATIENTS WITH MILD COGNITIVE IMPAIRMENT (MCI) AND DIABETES.* European Stroke Journal, 2023. **8**(2 Supplement): p. 25.

32. Xu, X., et al., *Cumulative Blood Pressure Load and Incident Dementia, Cognitive Function, and All-cause and Cardiovascular Deaths in Older Adults.* Heart Lung and Circulation, 2023. **32**(Supplement 3): p. S385EP-S386.

33. Keuss, S.E., et al., *Associations of beta-Amyloid and Vascular Burden with Rates of Neurodegeneration in Cognitively Normal Members of the 1946 British Birth Cohort.* Neurology, 2022. **99**(2): p. E129EP-E141.

34. Cleveland, M.L., *Preserving Cognition, Preventing Dementia.* Clinics in Geriatric Medicine, 2020. **36**(4): p. 585EP-599.

35. Wartolowska, K. and A. Webb, *Effect of past and concurrent elevated blood pressure on white matter hyperintensities: An analysis of the uk biobank cohort.* International Journal of Stroke, 2020. **15**(1 SUPPL): p. 16.

36. Flint, A.J. and K.S. Bingham, *Risk of vascular disease and cognitive function in very old adults.* International Psychogeriatrics, 2019. **31**(4): p. 443EP-446.

37. Levine, D., et al., *Blood pressure over the life course and later-life cognition in blacks, hispanics, and whites (BP-COG): Pooled cohort analysis of ARIC, CARDIA, CHS, FOS, and NOMAS.* European Stroke Journal, 2019. **4**(Supplement 1): p. 7.

38. Jenkins, L.M., et al., *HIGHER CUMULATIVE EXPOSURE TO BLOOD PRESSURE DURING YOUNG ADULTHOOD THROUGH MIDLIFE PREDICTS REDUCED LOCAL FUNCTIONAL HIPPOCAMPAL CONNECTIVITY AT REST IN MIDLIFE.* Alzheimer's and Dementia, 2018. **14**(7 Supplement): p. P39EP-P40.

39. Mayeda, E.R., et al., *Cumulative exposure to elevated blood pressure throughout adulthood and late-life dementia risk.* Alzheimer's and Dementia, 2017. **13**(7): p. P586EP-P587.

40. Al Hazzouri, A.Z., et al., *Racial/ethnic differences in the association of systolic blood pressure across midlife and late life on cognitive function: The multi-ethnic study of atherosclerosis.* Alzheimer's and Dementia, 2017. **13**(7): p. P196.

41. Richard, E., et al., *Prevention of dementia by intensive vascular care (prediva): A cluster randomized trial.* Alzheimer's and Dementia, 2016. **12**(7 Supplement): p. P342.

42. Derby, C.A., et al., *Vascular risk factors and cognitive function in midlife women: The study of women's health across the nation (SWAN).* Alzheimer's and Dementia, 2015. **11**(7 SUPPL. 1): p. P458EP-P459.

43. Jefferson, A.L., et al., *Low cardiac index is associated with incident dementia and Alzheimer's disease: The framingham heart study.* Alzheimer's and Dementia, 2014. **10**(SUPPL. 4): p. P678.

44. Yano, Y., et al., *Nocturnal blood pressure in young adults and cognitive function in midlife: The CARDIA study.* Circulation, 2014. **130**(SUPPL. 2).

45. Yano, Y., et al., *Long-term blood pressure variability though young adulthood and cognitive function in midlife; the coronary artery risk development in young adults (cardia)study.* Circulation, 2014. **129**(SUPPL. 1).

46. Yaffe, K., *The importance of risk factor modification over the lifecourse.* Neuropsychopharmacology, 2014. **39**(SUPPL. 1): p. S84.

47. Goncalves, A., et al., *Risk factors for blood transfusion in patients undergoing hip fracture surgery.* European Journal of Anaesthesiology, 2013. **30**(SUPPL. 51): p. 97.

48. Yaffe, K., et al., *Cardiovascular risk factors for cognitive function: Effects from early adulthood to mid-life.* Alzheimer's and Dementia, 2013. **9**(4 SUPPL. 1): p. P135.

49. Zhou, X. and S. Galea, *A study of blood pressure and change in cognitive function in elderly mexican Americans.* Pharmacoepidemiology and Drug Safety, 2011. **20**(SUPPL. 1): p. S244EP-S245.

50. Haan, M.N., et al., *Blood pressure, treatment and dementia risk in older Mexican Americans.* Stroke, 2011. **42**(3): p. e54EP-e55.

51. Levine, D.A., et al., *Impact of gender and blood pressure on post-stroke cognitive decline among older latinos.* Stroke, 2011. **42**(3): p. e56.

52. Gottesman, R.F., et al., *Blood pressure and white matter disease progression in a biethnic cohort.* Stroke, 2009. **40**(4): p. e129.

53. Richard, E., et al., *Prevention of dementia by intensive vascular care: Prediva.* Alzheimer's and Dementia, 2009. **5**(4 SUPPL. 1): p. 158.

54. Dettmers, C., et al., *Repetitive hypotensive episodes - Their contribution to vascular cognitive impairment.* Nervenarzt, 1997. **68**(8): p. 625EP-632.

55. Tarraf, W., et al., *Blood Pressure and Hispanic/Latino Cognitive Function: Hispanic Community Health Study/Study of Latinos Results.* JOURNAL OF ALZHEIMERS DISEASE, 2017. **59**(1): p. 31-42.

56. Xu, L.X.Y., Y. Yang, and D. Cui, *Association between blood pressure control status, visit-to-visit blood pressure variability, and cognitive function in elderly Chinese: A nationwide study.* FRONTIERS IN PUBLIC HEALTH, 2022. **10**.

57. Ernst, M.E., et al., *Long-Term Blood Pressure Variability and Risk of Cognitive Decline and Dementia Among Older Adults.* JOURNAL OF THE AMERICAN HEART ASSOCIATION, 2021. **10**(13).

58. Ou, Y.N., et al., *Longitudinal associations of cardiovascular health and vascular events with incident dementia.* STROKE AND VASCULAR NEUROLOGY, 2024. **9**(4): p. 418-428.

59. Franke, K., *Cumulative Blood Pressure Levels predictive for Dementia Risk.* AKTUELLE KARDIOLOGIE, 2022. **11**(05).

60. Jung, M.H., et al., *Relative importance of potential risk factors for dementia in patients with hypertension.* PLOS ONE, 2023. **18**(3).

61. Isrctn, *The 65 trial.* 2017.

62. Lamballais, S., et al., *血压、动脉僵硬度与认知的关系:基于2个人群(儿童和成人)的队列研究* 中华高血压杂志, 2018. **26**(12): p. 1154.

63. 刘茜, 闫中瑞, and 王海明, *血压变异性与认知功能障碍相关性的研究进展* 卒中与神经疾病, 2014. **21**(01): p. 56-58.

64. 刘小黎, 汪朝霞, and 黄炜婧, *高血压合并老年痴呆患者动态血压分析.* 中国煤炭工业医学杂志, 2011. **14**(04): p. 500-501.

65. 童宁, 程蕊, and 孙荣芝, *帕金森病患者血压变化与认知功能的研究进展.* 中国实用神经疾病杂志, 2024. **27**(09): p. 1179-1183.

66. 张旭明 and 王曦, *高血压对认知功能的影响.* 心血管病学进展, 2019. **40**(07): p. 977-981.

67. 张亚欣, 马丽娜, and 李耘, *老年高血压与认知功能的研究进展.* 疑难病杂志, 2014. **13**(06): p. 650-653.
